# Supplementary figures and images for: Barcode identification for single cell genomics
Source: BMC Bioinformatics. 2019 Jan 17;20:32. doi: 10.1186/s12859-019-2612-0 (PMC6337828; doi:10.1186/s12859-019-2612-0)

S1

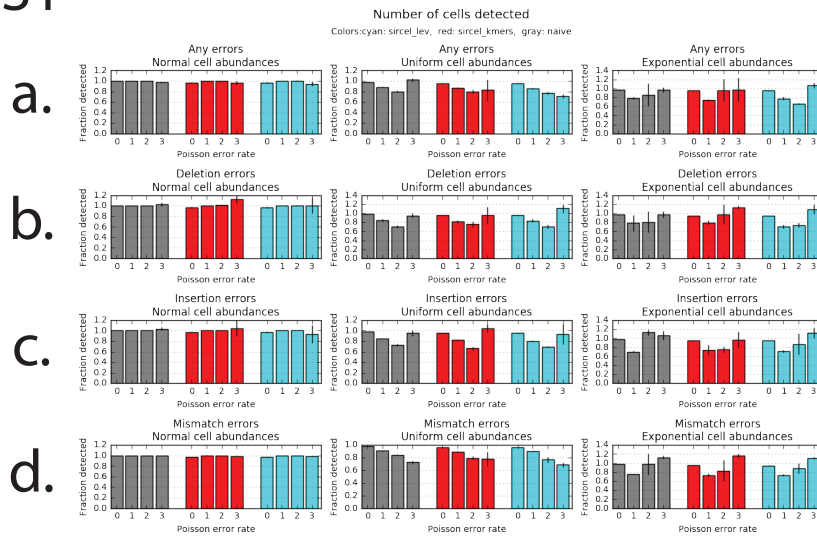

S2

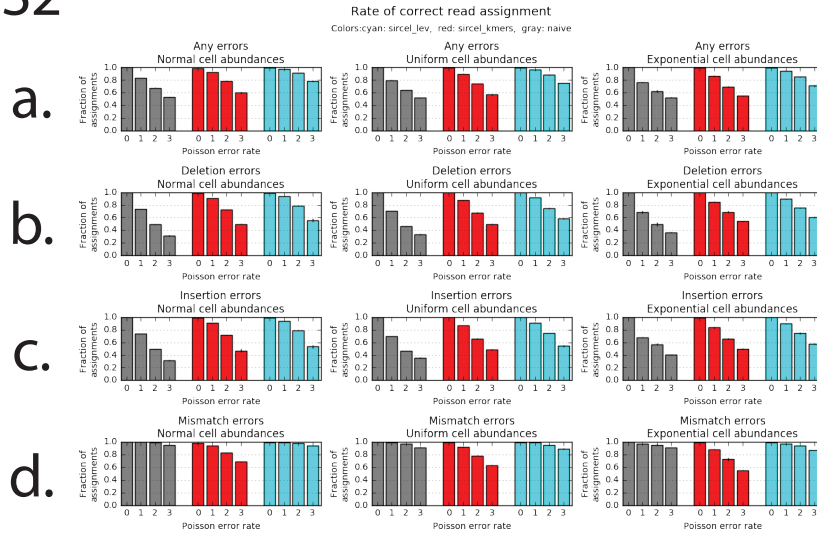

S3

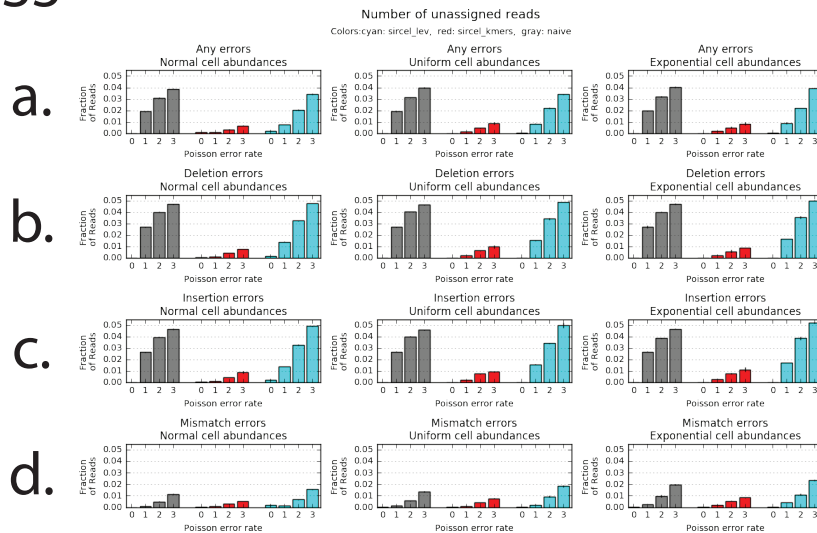

# S4

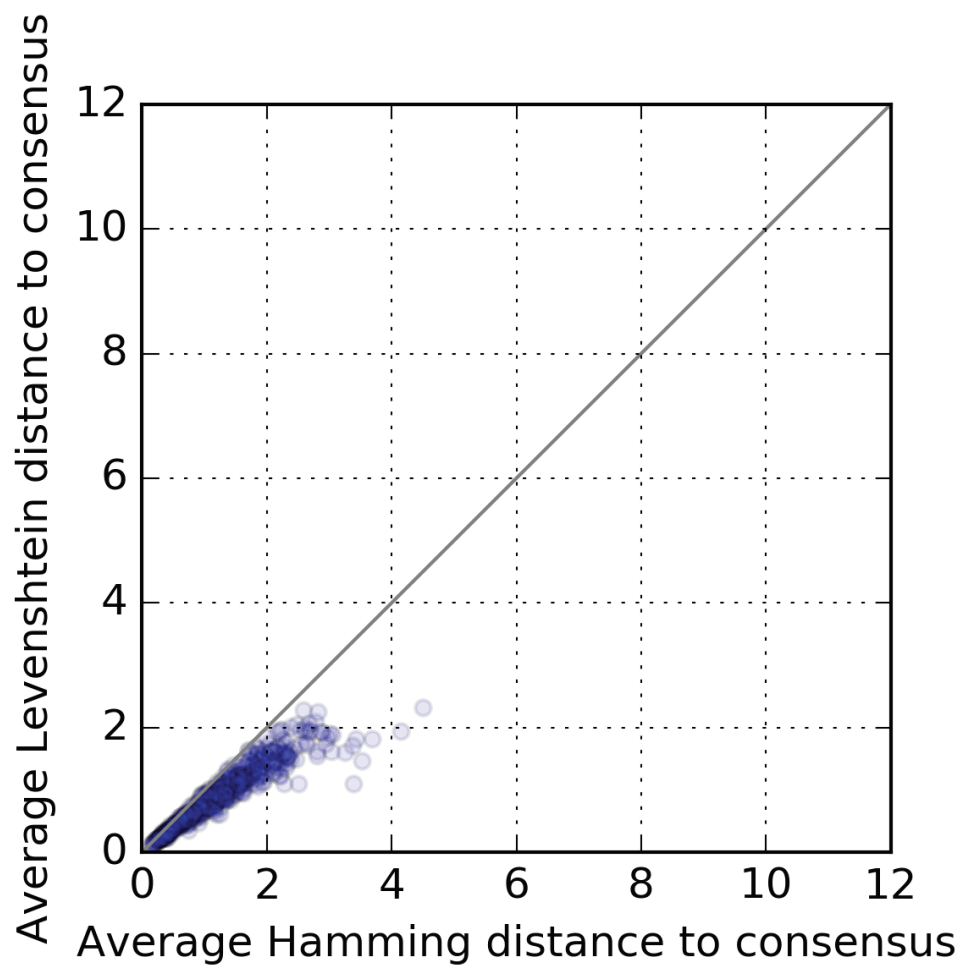

S5

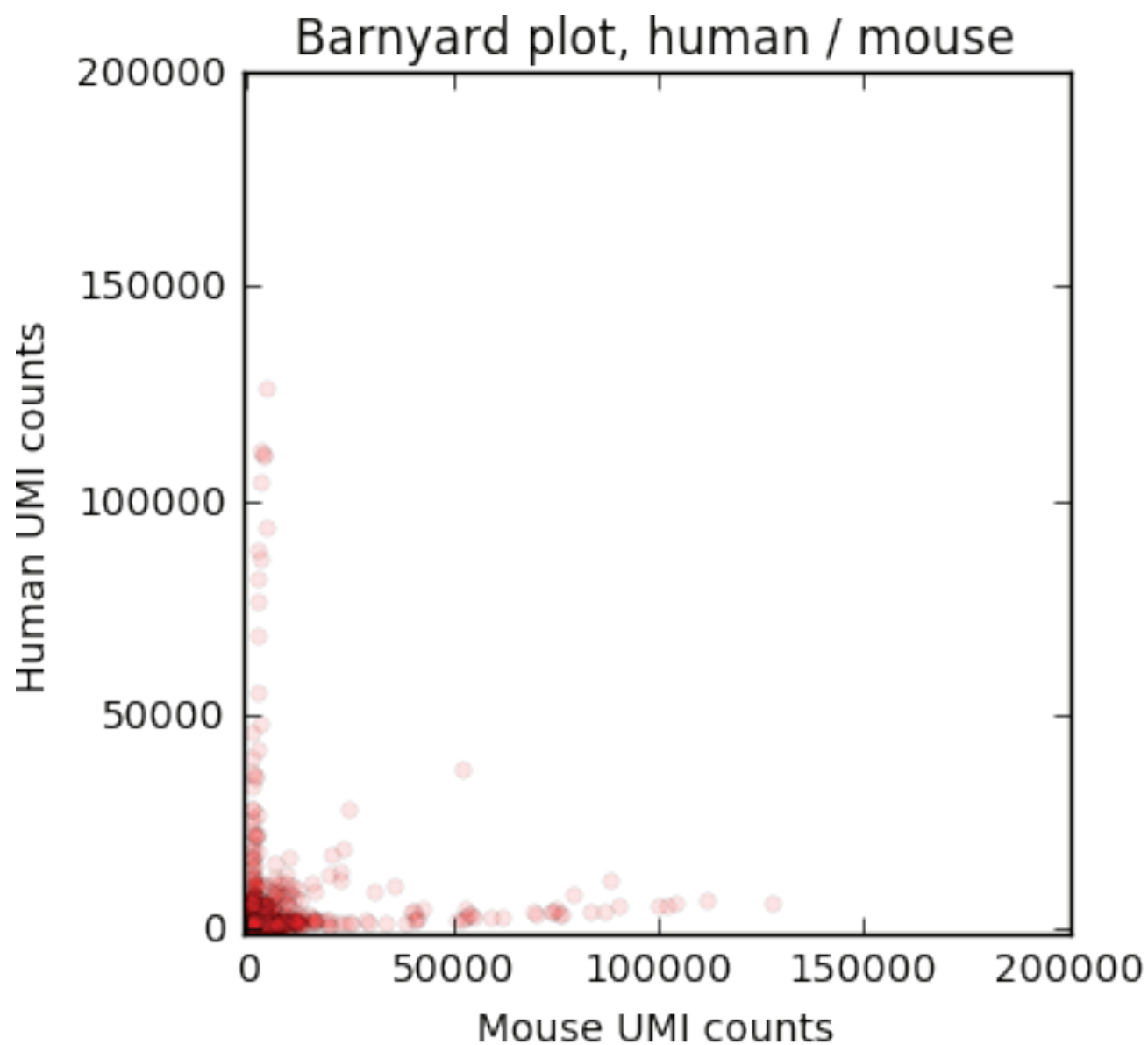

A.

## Cyclic kmer graphs for 10 barcode(s)

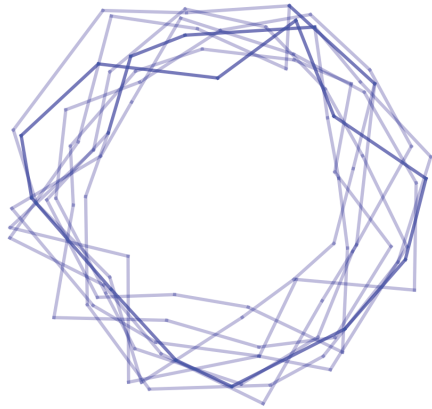

10 reads

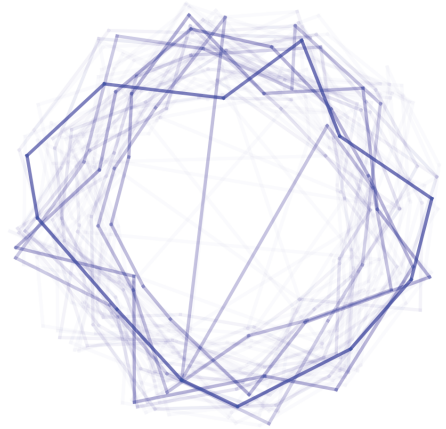

100 reads

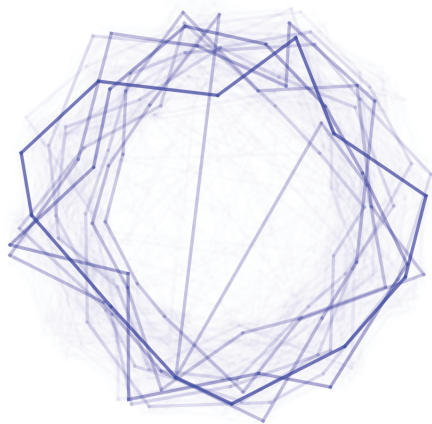

1000 reads

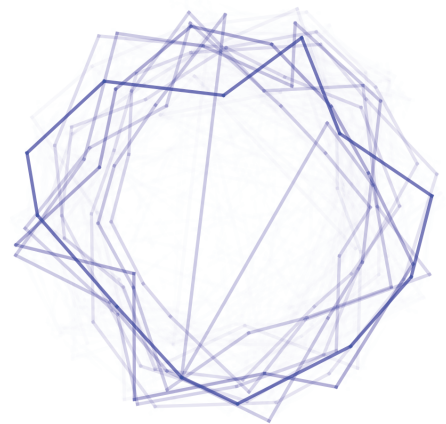

15000 reads

Supplement: Supplementary file 1 — Figure S1. Sircel can robustly identify the number of cells present in a dataset. We performed several simulations with error-prone reads. The number of errors per read, the type of errors, and the distribution of barcode abundances were all systematically varied. Performance was compared over three pipelines: a naïve approach (grey), using Sircel and k-mers (red) and using Sircel and Levenshtein distance (cyan). A. Any errors. B. Deletions. C. Insertions. D. Mismatches. Figure S2. Assigning reads to consensus barcodes depends on errors rate and barcode abundance distribution. Using the same simulations as before, the fraction of reads that were correctly assigned in each cell was quantified. We find that Sircel using Levenshtein distance performs the best. A. Any errors. B. Deletions. C. Insertions. D. Mismatches. Figure S3. Assigning reads to consensus barcodes by k-mer compatibility depends on errors rate and barcode abundance distribution. In the same simulations, the fraction of reads that could not be unambigiously assigned in each cell was quantified. A. Any errors. B. Deletions. C. Insertions. D. Mismatches. Figure S4. Indel errors are present in real data. We separated barcodes in a species mixing experiment from Seqwell (SRR5250839), and evaluated the Hamming and Levenshtein distances between each read and its consensus barcode. We find that Hamming distance is systematically larger than Levenshtein distance, indicating that the data contains indels. Figure S5. Species mixing with Seqwell data. We separated barcodes in a species mixing experiment from Seqwell and evaluated our ability to split reads by species. Figure S6. Circularized de Bruijn graph from real data. A de Bruijn subgraph was prepared from circularized reads that could be assigned assigned to 10 randomly selected barcodes from the Macosko et al. dataset is depicted here. Line transparency is proportional to the weight of each edge. (PDF 2486 kb) [file 12859_2019_2612_MOESM1_ESM.pdf]
